# Comparative Analysis and Dynamic Size Optimization of Aluminum and Carbon Fiber Thin-Walled Structures of a Railway Vehicle Car Body

**DOI:** 10.3390/ma18071501

**Published:** 2025-03-27

**Authors:** Alessio Cascino, Enrico Meli, Andrea Rindi

**Affiliations:** Department of Industrial Engineering, University of Florence, 50139 Florence, Italy; enrico.meli@unifi.it (E.M.); andrea.rindi@unifi.it (A.R.)

**Keywords:** railway vehicle design, dynamic optimization, structural optimization, carbon fiber composites, lightweight materials

## Abstract

In the context of modern railway engineering, the demand for lighter and more reliable vehicles has become a key objective for rolling stock manufacturers. Reducing energy consumption and minimizing environmental impact are driving the adoption of advanced materials and innovative design methodologies. This research activity focuses on a comparative analysis between aluminum and carbon fiber thin-walled structures used in railway vehicle car bodies. A high-fidelity finite element model (FEM) of a complete railway vehicle was developed to evaluate structural performance in compliance with European standards. Gaining deeper insights, one of the car body structures was isolated for a detailed dynamic analysis, enabling a comparative evaluation of the two materials. A structural dynamic size optimization process was applied to specific key components, aiming to maximize mass savings while maintaining mechanical integrity. The results exhibited an increase of approximately 10% in the first 10 car body eigenvalues, despite a mass reduction per unit of volume exceeding 30%, while largely preserving the nature of the eigenvectors. From a static perspective, both materials demonstrated good performance, with percentage differences below 20%. The optimization process highlighted significant potential for weight reduction in the analyzed structures. The findings highlight the critical role of optimization processes in streamlining design choices for lightweight structures. Moreover, they underscore the significant potential of high-performance carbon fiber materials in enhancing the efficiency and sustainability of railway vehicles. This study provides valuable insights for future research and practical applications in the field of lightweight railway vehicle design.

## 1. Introduction

The railway industry is undergoing a transformative shift driven by the need for more energy-efficient, lightweight, and environmentally sustainable solutions. Rolling stock manufacturers are increasingly focusing on advanced materials and innovative structural designs to achieve these objectives. Among the most critical aspects of railway vehicle design is the selection of materials for car body structures, as they significantly influence the overall weight, mechanical performance, and energy consumption of the vehicle [1,2]. Aluminum has long been a preferred choice for railway car bodies due to its favorable strength-to-weight ratio, corrosion resistance, and manufacturability [3]. However, recent advancements in composite materials, particularly carbon fiber-reinforced polymers (CFRP), have opened new possibilities for further weight reduction while maintaining or even enhancing structural integrity. The application of CFRP in railway vehicles remains an area of ongoing research, particularly concerning its mechanical performance, durability, and cost-effectiveness when compared to conventional materials like aluminum [4,5,6,7,8]. Moreover, it is essential to consider the mass of other structural components, such as the railway bogie frame and the bolster beam. For this reason, the combination of innovative materials and structural optimization techniques in the design of these complex components shows great potential, as highlighted in [9,10,11]. A crucial aspect of evaluating these materials is the use of high-fidelity numerical models, such as finite element models (FEMs), to simulate real-world conditions and assess their structural behavior. With reference to railway applications, three different types of composite panels were developed and evaluated to improve a railway vehicle component, undergoing an in-depth mechanical characterization to assess the properties of the lamina [12]. In comparison to traditional metallic materials, the weight of the modified component was about 72% of that of the aluminum alloy component, while still ensuring satisfactory tensile, fatigue, and impact resistance. The pultrusion manufacturing process was explored for producing mobile panels for medium-speed railway vehicles, aiming to replace steel panels with glass fiber-reinforced polymers (GFRP). The research found that the GFRP fabric pultrusion panel design effectively complied with the EN12663-1:2015 standard and project specifications, resulting in a 35.5% reduction in weight compared to the initial design [13]. Several factors, such as stiffness, strength, buckling, and thickness, were evaluated to determine their impact on choosing load-bearing sandwich panels for high-speed rail vehicles. Afterward, FE software was employed for optimization, leading to a 30% reduction in mass [14]. In [15], both experimental and numerical methods were used to evaluate the impact behavior of curved GFRP composites for railway vehicles. Furthermore, optimization processes were incorporated into sandwich structures to improve various properties. Topology, shape, and size optimization techniques were applied to enhance the ribs of a railway car body shell [16], aiming to achieve weight reduction. A similar approach was used for the redesign of the car body structure of a light rail vehicle. A dynamic size optimization methodology was developed to account for the modal behavior of the car body structure [17]. This methodology was then applied to an innovative car body structure incorporating a composite panel with a honeycomb core [18].

A thorough literature review confirms the limited number of applications involving the use of carbon fiber-reinforced polymers (CFRP) for the manufacturing of railway vehicle components. While CFRP has demonstrated remarkable mechanical properties, including high specific strength and stiffness, its implementation in railway structures remains relatively scarce due to several critical challenges. One of the primary obstacles is the necessity to comply with stringent European railway standards, which mandate rigorous testing and validation to ensure passenger safety and operational reliability. Unlike conventional metallic materials such as aluminum and steel, CFRP exhibits different failure mechanisms, including delamination, matrix cracking, and fiber breakage, which require specialized assessment methodologies to guarantee structural integrity under various loading conditions, including impact, fatigue, and fire resistance. Furthermore, cost-effectiveness remains a significant concern in the widespread adoption of CFRP in railway applications. Another crucial factor limiting CFRP adoption is the need for advanced numerical modeling and experimental validation to accurately predict its behavior under operational loads. Finite element models (FEMs) play a fundamental role in simulating real-world conditions, yet their accuracy depends on a deep understanding of material properties, boundary conditions, and load cases specific to railway environments. Existing studies have explored composite applications primarily for non-primary structures, such as interior panels and secondary load-bearing elements, yet the transition toward primary structural components, such as railway car bodies, requires further research and validation. Given these considerations, this research work aims to bridge the gap in knowledge by conducting an in-depth comparative analysis of aluminum and CFRP structures in railway vehicle applications. In order to gain deeper insights into their mechanical behavior, a comprehensive dynamic analysis was performed to evaluate their vibrational characteristics, modal behavior, and response to operational loads. A detailed finite element model of a complete railway vehicle was developed, followed by an isolated dynamic analysis of a specific car body structure. Additionally, a structural dynamic size optimization process was applied to key components, with the objective of maximizing weight savings while preserving mechanical performance. Through this approach, this research contributes to advancing the understanding of CFRP feasibility in railway structural applications while addressing the critical challenges associated with its implementation.

## 2. Methodology

The methodology consisted of the following steps:

(1) The FE model of the complete vehicle was required to be positively validated in accordance with the reference standard EN 12663-1:2015 [19]. Although loading conditions were consistently applied to the entire vehicle, it was essential for the reference car body to exhibit adequate mechanical performance, particularly regarding stress concentrations and modal behavior. Therefore, a comprehensive analysis of the vehicle was conducted for both materials.
(2) The reference car body was then isolated, incorporating all relevant components influencing the system’s dynamic behavior, like suspended equipment, modeled as concentrated masses. A modal analysis was conducted to assess its dynamic characteristics under free-free boundary conditions, observing potential differences in terms of eigenvalues and eigenvectors of the system. The first vibration frequency was subsequently considered as a constraint in the optimization process.
(3) The optimization approach was implemented on the reference car body with the aim of minimizing mass while ensuring the required dynamic performance, specifically in terms of the minimum vibration frequency.
(4) In order to complete the verification process, the innovative structure was subsequently subjected to a complete static analysis according to the reference standard.

## 3. Light Rail Vehicle Description

**Figure 1:**
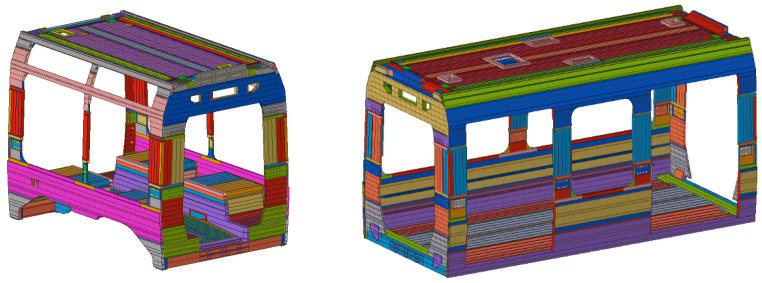
Not suspended car body on the (**left**), suspended car body on the (**right**).

The tram platform analyzed in this study consisted of a vehicle with five car bodies and three bogies. It was a monodirectional vehicle designed for urban operation and a low-floor vehicle. Such modern trams and certain railway trains introduce additional structural challenges due to their design requirements. The reduction in floor height necessitates modifications to the underframe, often leading to a more complex load distribution compared to conventional designs. In order to accommodate this, the structural layout typically incorporates reinforced sections around doorways and articulation points, where mechanical stresses are higher. Furthermore, the absence of a traditional high-floor structure often requires innovative solutions for housing essential systems, such as traction components and braking equipment, which are sometimes relocated to the roof or integrated into the bogies. These design choices aim to maintain structural integrity while maximizing passenger space and ensuring compliance with accessibility regulations. The vehicle was originally made entirely of aluminum alloy, with the end cabins constructed from composite material and all bogies made of high-strength structural steel. Focusing on the car body structures, which are the subject of this research, the vehicle featured two types, as shown in Figure 1. The first type, known as “suspended”, had greater longitudinal dimensions and was not directly connected to the bogie. This type of car body will be equipped with doors and will allow passengers to enter and exit. The second type, referred to as “not suspended”, was directly connected to the bogie system and, for this reason, exhibited different geometric characteristics concerning the lower frame. All car bodies were conceptually composed of long welded extrusions joined by welding. This approach is applied primarily to the roof assembly and the lower frame assembly, whose geometries are closely aligned with the most common structural designs of railway vehicles. However, a different approach was adopted for the intermediate assembly between these two, which was responsible for defining the window area and connecting the roof and underframe. In this case, the assembly was not carried out through welding but rather through riveting. A detailed model of the connection area was also developed, allowing for an in-depth verification of the structural performance.

### Structural Materials in Comparison

This research activity aimed to evaluate how the car body structure of a lightweight railway vehicle, made entirely or partially of carbon fiber-reinforced polymer, could alter its mechanical performance, from static and dynamic perspectives. Going into more detail about the aluminum alloys used (EN AW 6005 T6, EN AW 6106 T6), their main mechanical properties are shown in Table 1, according to the European standard EN 1999-1-1:2014 [20]. They are commonly used in structural applications due to their excellent balance of mechanical strength, corrosion resistance, and weldability. Both alloys belong to the 6xxx series, which is characterized by the presence of magnesium and silicon as primary alloying elements, leading to the formation of magnesium silicide (Mg_2_Si), which enhances their mechanical properties. EN AW 6005 T6 is widely used in transportation and structural applications due to its high strength and good extrusion properties. In the T6 temper, it undergoes a solution heat treatment followed by artificial aging, which significantly increases its yield strength and tensile strength. This alloy exhibits good resistance to atmospheric corrosion, making it suitable for railway vehicles, bridges, and marine applications. Its excellent machinability and weldability allow for the production of complex profiles with optimized weight-to-strength ratios, which is particularly beneficial in lightweight transportation applications. EN AW 6106 T6, while similar in composition to the previous one, has slightly different mechanical characteristics and is often chosen for applications requiring improved surface finish and formability. This alloy also benefits from high strength and corrosion resistance, but its primary advantage lies in its enhanced resistance to stress corrosion cracking and fatigue. Due to these properties, EN AW 6106 T6 is frequently used in components exposed to cyclic loads, such as railway and automotive structures. Additionally, its good anodizing response allows for improved aesthetic and protective surface treatments.

There are many possible solutions when it comes to carbon fiber, with highly variable mechanical performance and applications. T300 carbon fiber has been investigated during this research activity [21,22]. T300 carbon fiber is a high-performance material widely used in aerospace, automotive, and railway applications due to its excellent strength-to-weight ratio, high stiffness, and good fatigue resistance. It is commonly used in combination with epoxy resins to create carbon fiber-reinforced polymer (CFRP) composites, which offer superior mechanical properties and environmental resistance. When combined with epoxy resin, T300 carbon fiber exhibits enhanced durability, impact resistance, and thermal stability. Epoxy resins provide excellent adhesion to carbon fibers, ensuring efficient load transfer and structural integrity. This combination is widely employed in railway vehicle structures, such as lightweight car bodies and structural reinforcements, to reduce weight while maintaining high mechanical performance. T300/epoxy composites are particularly advantageous for applications requiring low weight, high strength, and resistance to corrosion. These materials are increasingly used in modern transportation systems to improve energy efficiency, reduce emissions, and extend service life. Table 2 summarizes the material properties.

## 4. FE Dynamic Optimization: Results and Discussion

In this section, all the fundamental steps from a numerical perspective will be described for an effective comparison between the two proposed solutions.

### 4.1. FE Model and Analysis Settings

The FE model of the vehicle was a high-level representation of the system. It counts about 6 million nodes and 5.6 million elements. The structure of the car body, including all the subassemblies, has been modeled using a two-dimensional mesh including SHELL elements of QUAD4 type, all in first order. The average size was about 20 mm, with a local refinement of the grid useful to increase the quality and reliability of the stress assessment. All the rivets were modeled with a simplified approach, using two rigid multi-node elements of type RBE2 combined with a proper 1D beam element, representative of the real section of the component All the main equipment connected to the vehicle was represented by exploiting concentrated masses (CONM2 type) connected to the structure through RBE3 elements. The railway vehicle car body shell exhibited reduced thicknesses, effectively represented by the numerical formulation describing the mechanical behavior of shell elements. The constraint conditions were consistently defined by an isostatic configuration, where the car body was vertically supported at the secondary suspension, laterally constrained at the side pads, and longitudinally restrained at the rear buffers of the cabin. All simulations were carried out using the same computer, which had the following characteristics: Intel(R) Xeon(R) CPU E5-2643 v4 @ 3.40GHz, RAM 32 GB. According to the EN 12663-1 standard, this vehicle fell under the P-V category, which includes passenger vehicles, specifically tramway vehicles. The standard requires to test the complete model of the vehicle. This classification establishes the various load conditions required for system testing, as shown in Table 3. All the masses used for the numerical assessment of the vehicle were referred to UNI EN 15663:2019 standard [23], which defines the terminology and classification of railway vehicle masses, providing guidelines for their determination. This standard is essential for ensuring a consistent approach to mass classification, facilitating comparability across different railway systems, and contributing to operational safety and efficiency. It outlines specific mass categories, including tare mass (C0), operational mass (C2), and maximum laden mass (C4), which are crucial for the design, maintenance, and management of rolling stock.

To conclude, as deeply described above, the entire methodology used was strictly based on the European reference standards which are regularly adopted by major manufacturers and builders of rolling stock. In this context, experimental validation of the numerical could be a subsequent step to verify local concentrations of potentially dangerous stress observed during the finite element numerical analysis. However, experimental support is never defined as a priori in the development of a finite element model for a railway vehicle car body but is always set afterward to check where necessary.

### 4.2. Static Analysis of the Complete Vehicle

**Figure 2:**
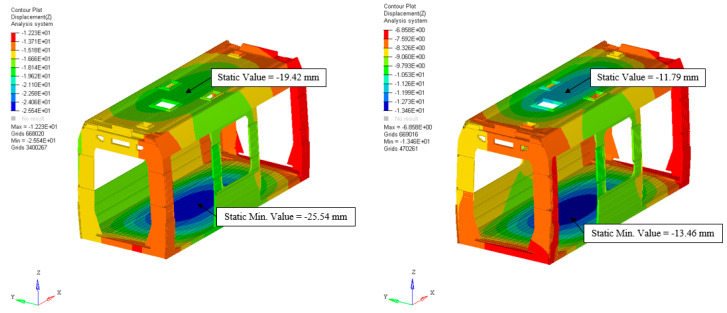
Aluminum vs. T300/epoxy: roof and underframe deflection.

As specified in the previous section, the static analysis, performed in accordance with the load conditions outlined in the European standard, must always be conducted on the complete vehicle, also considering all major car body-to-car body interfaces and the relative motions involved. Two simulations were carried out, allowing for a comparison of the performance of the two materials. The selected reference load case corresponds to the one listed in Table 3 with index 1. This load case assumes the presence of six people per square meter, each weighing approximately 70 kg, and has been identified as the most critical in terms of structural resistance and deflection. For this reason, it will be used as a reference for the analysis of the subsequent results, which were evaluated on the suspended car body. In terms of purely vertical deflection, the analysis focused on the two most critical structural elements: the roof and the underframe. Both exhibited global flexure, with the highest recorded deflection values observed in the structure. The difference between the two materials is significant, with a reduction of approximately 47.3% in deflection on the underframe and about 39% on the roof. These results confirm the excellent flexural performance offered by the T300/epoxy material. From an engineering and structural design perspective, these findings highlight the potential for developing geometric solutions that further enhance the material performance while minimizing its contribution to overall mass. Figure 2 illustrates the results just described.

**Figure 3:**
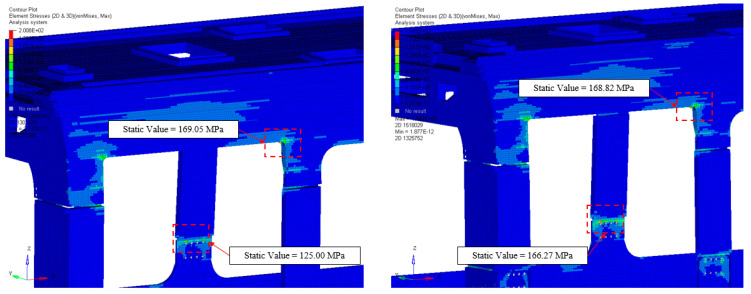
Aluminum vs. T300/epoxy: stress concentrations (door and uprights areas).

With reference to the stress values calculated according to Von Mises criteria, the analysis focused on the two most critical areas: the connection of the uprights and the structure housing the door system. In railway vehicles, stress concentrations are typically observed in the window and door areas, and these results confirm this trend. Focusing first on the upright connection area, an increase in stress of approximately 24.6% was observed in the carbon fiber structure. This result is entirely reasonable, as the higher stiffness of the material leads to greater resistance to local flexural effects, resulting in higher stress levels. However, these values remain well within the admissibility limits, as shown in Table 2. Conversely, the same reasoning does not apply to stress concentrations in the door area. As observed in the results, the variation in stress levels is practically negligible. This is because, in this region, the geometry of the car body structure plays a dominant role compared to the material class used. For this reason, both from a static and fatigue analysis perspective, an in-depth experimental investigation using appropriately positioned strain gauges would certainly be required in this area. This result, shown in Figure 3, is reasonable and remains well within the admissibility limits of the material parameters. The static analysis, examined and discussed in its most critical configuration, has therefore yielded a positive outcome.

### 4.3. Modal Analysis of the Single Car Body

**Figure 4:**
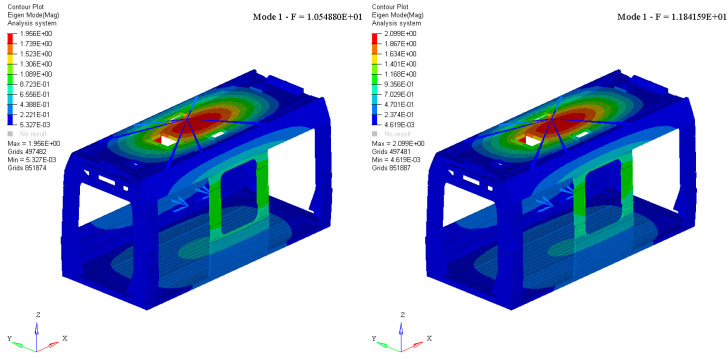
Aluminum vs. T300/epoxy: mode shape number one (undeformed view).

**Figure 5:**
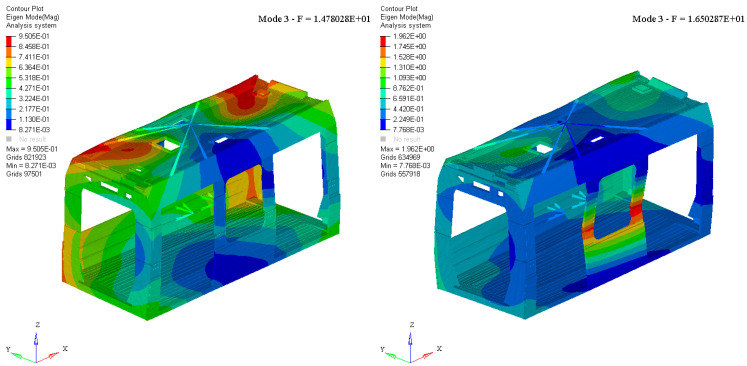
Aluminum vs. T300/epoxy: mode shape number three (deformed view).

The evaluation of the modal behavior of a railway car body is crucial for ensuring structural integrity, passenger comfort, and overall vehicle performance. A thorough analysis of the car body’s natural frequencies and mode shapes allows for the identification of potential resonance phenomena that could amplify vibrations and compromise ride quality. In particular, it is essential to achieve an effective decoupling between the car body modal response and the dynamic motions of the bogies. If strong coupling occurs, undesired vibrational interactions can lead to excessive accelerations, increased fatigue loads, and reduced service life of structural components. Properly tuning the modal characteristics of the car body, through optimized design and material selection, helps mitigate these effects and enhances the stability and safety of the railway vehicle. The modal behavior of the single car bodies was evaluated, comparing the first mode shapes in free-free conditions, to assess the effect of changes in the material. Modal analysis was carried out on all the car bodies. Figure 4 and Figure 5 show the comparison of two mode shapes: Mode 1 and Mode 3.

Both modes were identified as fundamental car body modes. Mode 1 exhibited a typical bending nature, primarily involving the roof of the car body structure. This result was confirmed for both material types, demonstrating a nearly unchanged dynamic behavior in terms of mode shape. The reference eigenvalue showed a variation of approximately 11%, a still acceptable increase that further confirms the complete decoupling from potential dynamic bogie modes. Mode 3 exhibited a more complex vibrational behavior, with no clearly distinguishable nature. In both material configurations, it was a global mode involving the entire car body shell. However, this behavior led to different effects between the two tested solutions. In the aluminum case, the car body moved globally, with the maximum displacement recorded at the roof extremities. The mode shape appears to be predominantly torsional. Conversely, in the T300/epoxy configuration, the vertical structure of the door pillars experienced a significant bending effect compared to the rest of the car body, resulting in a predominant flexural behavior. Notably, of further importance is the variation in terms of eigenvalue, approximately equal to 10%.

**Figure 6:**
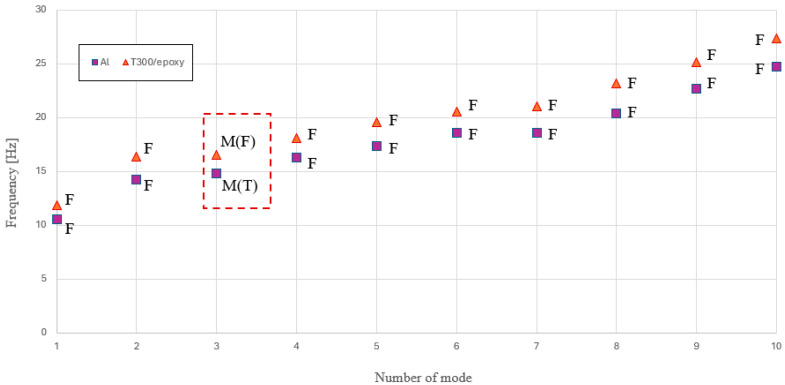
Modal analysis overview: eigenvalues and eigenvectors.

The graph in Figure 6 provides an in-depth understanding of the modal behavior of the analyzed car body and how it has changed. The legend indicates the marker associated with each material. Additionally, letters have been introduced to help interpret the nature of the considered vibration mode: F stands for flexural, T for torsional, and M for mixed. The combination of these letters for Mode 3, confirming the findings shown in Figure 5 and previously described in detail, indicates a mode of vibration with an undefined nature, thus classified as mixed, but with a predominant dynamic behavior, either flexural or torsional. In a railway vehicle, the rigid-body vibration modes of the car body occur at low frequencies and are primarily influenced by the suspension system and the interaction with the track. These modes include vertical bounce, lateral sway, roll, pitch, and yaw, typically ranging from fractions of a Hz to a few Hz. As the frequency increases, the behavior of the car body transitions from rigid-body motion to flexible-body vibrations. At higher frequencies, typically in a range between 8 and 10 Hz, structural deformation becomes significant, leading to flexible modes such as bending and torsional vibrations of the car body. These flexible modes are critical for passenger comfort and structural integrity, as they can influence ride quality and dynamic responses under operational conditions. For the tested car body, the first mode of vibration was up to 10 Hz, allowing a safe decoupling from dangerous bogie motions, such as hunting motion, which is generally present around 6 Hz. The graph reveals a global shift of approximately 10% in terms of eigenvalues. Furthermore, a clear predominance of flexural behavior is observed in the first 10 vibration modes of the structure, for both materials used. Only Mode 3 exhibits a variation—although not sharply defined—in the nature of the mode. This result clearly highlights the significant impact of the car body shell geometry on its dynamic behavior. Based on this finding, even though it is beyond the scope of the present study, a thorough assessment of the structure buckling behavior would be necessary to prevent the occurrence of dangerous local buckling phenomena.

### 4.4. Dynamic Size Optimization of the Single Car Body

The optimization process focused on the roof assembly. The main hypothesis of the procedure was to maintain the geometry of the tested components unchanged while analyzing two different materials to compare their mechanical performance. Consequently, two distinct optimization processes were carried out, each corresponding to a specific material class. The aim of the size optimization process is to determine the minimum thickness that satisfies both the objective function and the design constraints of the problem. The formulation of a structural optimization problem is outlined in [24] and summarized as follows:

(1) $$Find\;d\in R^{n},y\in R^{l}$$

(2) to minimize   f(d,y)

(3a) $$subject\;to\;K\left(d\right)y=f$$

(3b) $$c_{j}\left(d,y\right)\leq 0;j=1,\ldots ,z$$

(3c) $$d_{L}\leq d\leq d_{U}$$

**Figure 7:**
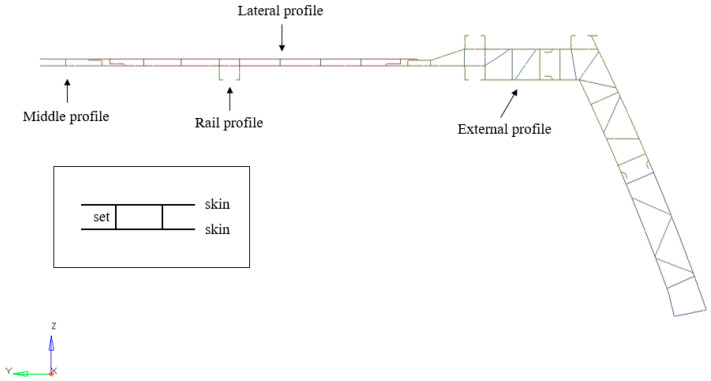
Design variables of the optimization problem (roof assembly).

Starting from Equation (1), $d\in R^{n}$ represents the design variable vector, while $y\in R^{l}$ is the vector of nodal displacements of the grid. The two exponents n and l indicate the number of design variables and the total number of degrees of freedom of the system, respectively. K is the stiffness matrix and f is the vector of external loads. f represents the objective function of the optimization problem, $c_{j}$ is the constraint function (number of constraints equal to z). In Equation (3a) is described the governing equation of linear finite element analysis, $K\left(d\right)y=f.$ In Equation (3c), $d_{L}$ and $d_{U}$ are the lower bound (L) and the upper bound (U) of the vector, which define the range of variation of the design variables values. The process aimed to minimize mass while ensuring that the minimum vibration frequency of the complete car body remained at or above 10.5 Hz. This threshold corresponds to the lowest value identified in the modal analysis of the two structures made up of different materials. The main components whose thicknesses represented the design variables, are illustrated in Figure 7, including both skins and sets. A minimum thickness of 1 mm was set for all components to assess the extent to which the material could still be utilized. This consideration was based on recent advancements in aluminum extrusion technology (ALE), which allow for the production of increasingly thinner profiles. Table 4 provides a detailed summary of the structural optimization results for the selected components of each of the two chosen materials. It also includes the initial thickness values and the allowed variation ranges.

The optimization results confirmed the findings previously observed in both the static and modal analysis. The T300/Epoxy material, due to its excellent mechanical performance, which was fully exploited under the loading conditions of the car body structure, enabled the design of slimmer yet equally resistant structures. This resulted in a dual mass-saving advantage, as the material itself had a density approximately 35% lower than that of aluminum. Additionally, it was confirmed that the first vibration mode maintained its flexural nature, with the main deformation still concentrated in the roof and underframe assemblies. The reduction in thickness certainly leads to a decrease in mass. However, the optimization process is specifically aimed at keeping the first natural frequency of the system sufficiently high, thereby ensuring an optimal stiffness-to-mass ratio while also maintaining mechanically compliant performance.

**Figure 8:**
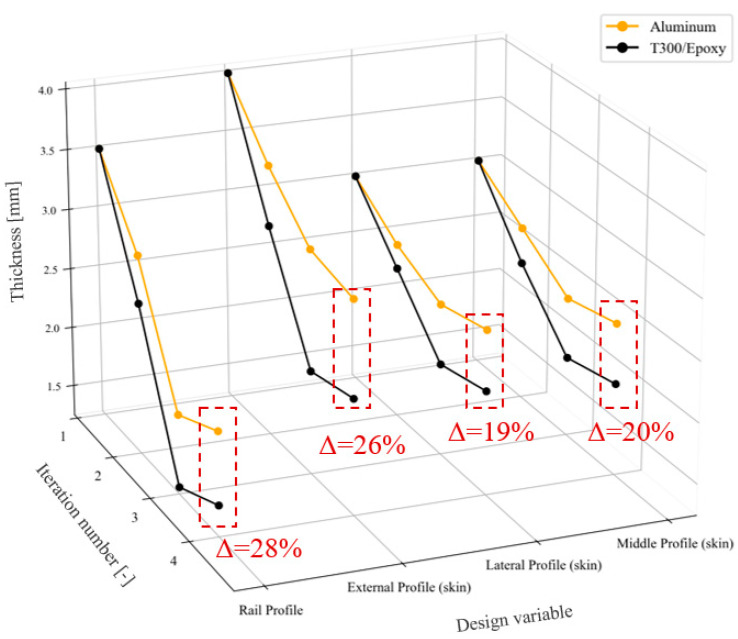
Evolution of thickness values during optimization and final percentage variations on thickness.

Finally, the trend of the structural optimization process applied to some of the design variables, selected as a reference to highlight this result, is summarized in Figure 8. The process trend for the two tested materials shows a consistent pattern. In particular, in the case of T300/Epoxy, thanks to its stiffness characteristics, a rapid decrease in thickness was observed for the analyzed components, more prominently than for aluminum. For the latter, the optimization algorithm quickly converges to a plateau, reaching the optimal value according to the analyzed parameters imposed on the problem. In order to ensure a more meaningful data visualization, the percentage deviation between the optimized thicknesses obtained with the two different materials is shown in red. These values all fall within a range of 20% to 30%, once again highlighting the significant differences in terms of mass that can be achieved while maintaining the same geometry. In terms of stress, the new performance was found to be compliant with the permissible material values.

## 5. Conclusions

This research work conducted a comparative analysis between aluminum and carbon fiber thin-walled structures used in railway vehicle car bodies. A lightweight P-V category vehicle was analyzed in accordance with the reference standard for verifying the performance of railway vehicle car body shells, which is extensively described in the dedicated chapters. The analysis carried out provided a deeper understanding of the effects of a significant material change in the construction of these complex structures. Aluminum (EN AW 6005 T6, EN AW 6106 T6) was compared with T300/Epoxy, a material composed of properly treated carbon fiber. This initial research phase constrained the vehicle geometry, analyzing only the performance of the two materials under the same conditions through a modal analysis in free-free conditions (typical in the railway field), a static structural analysis, and a dynamic size optimization process. The vibrational modal analysis showed a general increase in the system eigenvalues by approximately 10%, despite the density variation between the two materials exceeding 30%. This aspect is certainly also attributed to the increased stiffness provided by T300/Epoxy. Despite this significant change, the nature of the modal shapes remained unaltered, maintaining an almost entirely flexural behavior at least for the first 10 analyzed body modes. From a static perspective, both materials exhibited satisfactory stress performance within the allowable limits. The results in terms of deflection, particularly concerning the roof assembly and the underframe assembly, showed a significant variation exceeding 20%. Finally, the optimization process revealed a general reduction in the analyzed design variables for both materials. This highlighted potential mass reduction opportunities, which could be further enhanced by using low-density materials such as T300/Epoxy. In addition, it is crucial to emphasize that this significant advantage would be supported by the preservation of the modal shape characteristics of the structure. This would enable substantial time savings in ride dynamics analysis during the vehicle development phase, without the need to seek complex solutions to prevent coupling between structural modes of the body and bogie or other structurally and vibrationally critical modes during operation. In conclusion, the two analyzed materials exhibited good mechanical performance and similar dynamic characteristics. This result opens up significant opportunities and future developments in the use of innovative materials, such as carbon fiber-based composites. The goal is to innovate the geometries of lightweight railway vehicles to make them suitable for manufacturing with less conventional production techniques. This approach would help reduce production costs, which are currently higher than those of metallic materials, while simultaneously achieving a significant reduction in vehicle mass. As a result, energy consumption would decrease, benefiting both efficiency and the environment.

## Figures and Tables

**Table 1:** Mechanical properties of the aluminum alloys (EN 1999-1-1:2014).

Material	Density	Young’s Modulus	Proof Strength (0.2%)	Ultimate Tensile Strength	Proof Strength (0.2%)	Ultimate Tensile Strength	Poisson’s Ratio
			Base Material		Weld Material		
	[kg/m^3^]	[N/mm^2^]	[N/mm^2^]		[N/mm^2^]		[-]
EN AW 6005 T6	2700	70,000	215	255	115	165	0.30
EN AW 6106 T6	2700	70,000	200	250	95	160	0.30

**Table 2:** Mechanical properties of the T300/epoxy carbon fiber.

Material	Density	Young’s Modulus	Ultimate Tensile Strength	Ultimate Compressive Strength	Elongation at Break
	[kg/m^3^]	[N/mm^2^]	[N/mm^2^]	[N/mm^2^]	[%]
T300/epoxy	1760	135,000	1860	1460	1.5–2.0

**Table 3:** Loading conditions according to EN 12663-1:2015.

Index	Type of Load	Load Formula
1	Vertical	Vertical load = 1.3 × g × C4
2	Compressive on buffers, C0 condition	Vertical load = C0 × g Longitudinal force = 200,000 N
3	Compressive on buffers, C4 condition	Vertical load = C4 × g Longitudinal force = 200,000 N
4	Tensile on drawbar, C0 condition	Vertical load = C0 × g Longitudinal force = 55,000 N
5	Compressive on drawbar, C0 condition	Vertical load = C0 × g Longitudinal force = 100,000 N

**Table 4:** Optimization results: thickness variation of design variables.

Design Variable	Starting Thickness		Range of Thickness Variation (Max–Min)	Optimized Thickness (Aluminum)	Optimized Thickness (T300/Epoxy)
[-]	[mm]		[mm]	[mm]	[mm]
Middle profile	skin	3.0	3.0–1.0	2.5	2.0
	rib	3.0	3.0–1.0	1.5	1.2
Lateral profile	skin	3.0	3.0–1.0	2.6	2.1
	rib	3.0	3.0–1.0	2.6	2.0
External profile	skin	4.0	4.0–1.0	3.0	2.2
	rib	3.0	3.0–1.0	2.2	1.6
Rail profile	3.5		3.5–1.0	2.1	1.5

## Data Availability

The original contributions presented in this study are included in the article. Further inquiries can be directed to the corresponding author.
